# The value of shoe size for prediction of the timing of the pubertal growth spurt

**DOI:** 10.1186/1748-7161-6-1

**Published:** 2011-01-20

**Authors:** Iris Busscher, Idsart Kingma, Frits Hein Wapstra, Sjoerd K Bulstra, Gijsbertus J Verkerke, Albert G Veldhuizen

**Affiliations:** 1 University Medical Center Groningen, University of Groningen, Department of Orthopaedics, Hanzeplein 1, 9713 GZ Groningen, The Netherlands; 2 Research Institute MOVE, Faculty of Human Movement Sciences, VU University Amsterdam, van der Boechorstraat 9, 1081 BT Amsterdam, The Netherlands; 3 University Medical Center Groningen, University of Groningen, Department of Biomedical Engineering, Antonius Deusinglaan 1, 9713 AV Groningen, The Netherlands; 4 University of Twente, Department of Biomechanical Engineering, Drienerlolaan 5, 7522 NB Enschede, The Netherlands

## Abstract

**Background:**

Knowing the timing of the pubertal growth spurt of the spine, represented by sitting height, is essential for the prognosis and therapy of adolescent idiopathic scoliosis. There are several indicators that reflect growth or remaining growth of the patient. For example, distal body parts have their growth spurt earlier in adolescence, and therefore the growth of the foot can be an early indicator for the growth spurt of sitting height. Shoe size is a good alternative for foot length, since patients can remember when they bought new shoes and what size these shoes were. Therefore the clinician already has access to some longitudinal data at the first visit of the patient to the outpatient clinic.

The aim of this study was to describe the increase in shoe size during adolescence and to determine whether the timing of the peak increase could be an early indicator for the timing of the peak growth velocity of sitting height.

**Methods:**

Data concerning shoe sizes of girls and boys were acquired from two large shoe shops from 1991 to 2008. The longitudinal series of 242 girls and 104 boys were analysed for the age of the "peak increase" in shoe size, as well as the age of cessation of foot growth based on shoe size.

**Results:**

The average peak increase in shoe size occurred at 10.4 years (SD 1.1) in girls and 11.5 years (SD 1.5) in boys. This was on average 1.3 years earlier than the average peak growth velocity of sitting height in girls, and 2.5 years earlier in boys. The increase in shoe size diminishes when the average peak growth velocity of sitting height takes place at respectively 12.0 (SD 0.8) years in girls, and 13.7 (SD 1.0) years in boys.

**Conclusions:**

Present data suggest that the course of the shoe size of children visiting the outpatient clinic can be a useful first tool for predicting the timing of the pubertal growth spurt of sitting height, as a representative for spinal length.

This claim needs verification by direct comparison of individual shoe size and sitting height data and than a step forward can be made in clinical decision making regarding adolescent idiopathic scoliosis.

## Background

In adolescent idiopathic scoliosis in particular it is highly important to know when the peak growth velocity of spinal length takes place. Since a close relationship exists between the spinal growth of the patient and the angle progression of the idiopathic scoliosis, it is essential to know the timing of the pubertal growth spurt in order to determine the optimum treatment strategy for the individual child [1-4]. However, exact spinal length is hard to obtain and therefore sitting height can be a representative alternative.

It is still highly difficult to predict when the individual child will have his or her pubertal growth spurt. 95% of the girls experience their peak growth velocity between ages ten and fourteen, and 95% of the boys between age twelve and sixteen [5-7]. This range is too wide to be able to make an accurate prediction in the individual patient.

It is known that different parts of the body each have their own typical growth pattern. Cameron et al.[8], Dimeglio[9], and Welon and Bielicki[10] all confirm the distal-to-proximal growth gradient as described earlier by Tanner[11], meaning that more distal body parts have their pubertal growth spurt earlier in adolescence. Furthermore, it is known that the sequence of growth spurts of different body parts is similar in individual children, regardless of being an "early" or "late" maturer[10]. Therefore, the timing of the growth spurt of foot length could be an early indicator for the timing of the growth spurt of sitting height [12].

For an accurate determination of the timing of the peak growth velocity of foot length, longitudinal growth data are needed. However, during a first visit to the outpatient clinic patients usually don't have information concerning their foot lengths over the last years. Therefore the clinician can not make an accurate assessment at that moment, as would be preferable. To overcome this lack of information concerning the longitudinal growth of the foot, it is useful to have a representative alternative for foot length. This could be the shoe size. Pilot work for present study in the orthopaedic outpatient clinic confirmed that patients and their parents remember to a large extent when they bought new shoes and what size these shoes were. This could therefore be a very easy, first clinical tool that can help in determining the timing of the growth spurt of the foot, and therefore in predicting the timing of the growth spurt of sitting height.

Information concerning longitudinal increases in shoe size is lacking and therefore the aim of this study was to describe the increase in shoe size during adolescence. It was hypothesized that an earlier "peak increase" in shoe size would occur in comparison to the general timing of the peak growth velocity of sitting height, and that therefore the increase in shoe size could be an early indictor for the timing of the pubertal growth spurt.

## Methods

Longitudinal data of shoe sizes were collected in two large shoe shops in the Netherlands from 1991 to 2008. Data were collected in a similar manner in the two shops. Data were collected each time a client visited the shoe shop. Each client's bare left and right foot were measured individually while standing full weight bearing. The client stood in the calliper with the back of the heel against the stationary arm, and the movable arm was brought into contact with the tip of the longest toe. The shoe size was measured to the nearest half-size, and an average was taken for both feet. Shoe size was expressed in European standards. For translation to English and American sizes see Table 1.

**Table 1:** Translation of shoe sizes for different regions.

Europe	UK	USA	
		**Male**	**Female**

36	3		4.5

36.5	3.5		5

37	4		5.5

37.5	4.5		6

38	5	5.5	6.5

38.5 - 39	5.5	6	7

39 - 39.5	6	6.5	7.5

40	6.5	7	8

40.5	7	7.5	8.5

41 - 41.5	7.5	8	9

42	8	8.5	9.5

42.5	8.5	9	10

43	9	9.5	

43.5 - 44	9.5	10	

44 - 44.5	10	10.5	

45	10.5	11	

46	11	11.5	

46.5	11.5	12	

UK = United Kingdom, USA = United States of America

The shoe sizes used for data-analysis were the standard measured sizes, and not the size of the shoes which were sold to the clients.

The age at the time of measurement was determined by subtracting the date of birth from the date of the measurement.

The data were anonymized by an external organisation before transfer to the researchers.

**Figure 1:**
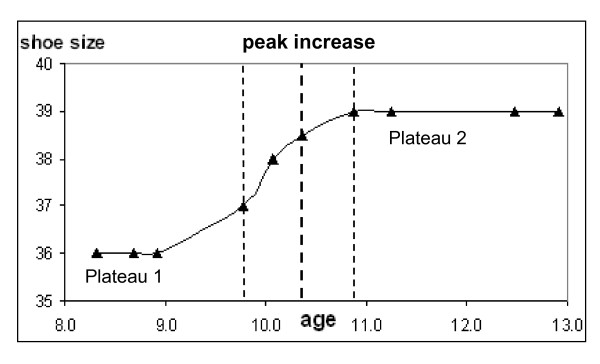
**Example of a growth curve of the shoe size of a girl**. The start and end of the peak increase of the shoe size are shown as dotted lines. The age at peak increase is shown as the fat dotted line. Age is expressed in years, shoe size in European standards.

The "period of peak increase" in shoe size was defined as the shortest period in which the shoe size increased by two whole sizes. The age of the peak increase was defined as the age in the middle between the start-age and the end-age of this period (peak increase in Figure 1). The average magnitude of the increase was calculated in shoe sizes per year.

A customized program in Matlab^® ^(Mathworks, Natick MA, USA) was used to plot the data of each individual client, and the results were checked visually for outliers. The period of peak increase was calculated by the program, as well as the age of the peak increase and the peak velocity of shoe size increase.

Secondary outcome measures were related to the cessation of foot growth. The age at the end of foot growth was determined in the individual graphs as the age from where the shoe size did not increase any further. This plateau period should at least continue for 1 year for the client to be included in the data-analysis of the plateau phase (plateau 2 in Figure 1). Furthermore, the start-age of the plateau phase should be higher than the age of the peak growth velocity. In this way temporary and short plateau phases, or phases which occurred before the peak growth, were excluded (plateau 1 in Figure 1).

Clients who had more than 4 measurements above the age of 8 years, as well as a follow up time of more than two years were selected. Furthermore the last measurement of shoe size should be above 12 years in girls and 13 years in boys. Only data above 8 years were used for analysis of the peak increase in shoe size since it was not expected that the growth spurt would occur earlier in healthy children, and to exclude boys and girls with precocious puberty. The selection of the clients was performed to make sure the pubertal growth spurt in shoe size was not missed. For example, if a client had data until age 10 or 11 years, the Matlab program could still calculate a shortest period in which the shoe size increased two sizes. However, this would result in a "false growth spurt" which would not represent the actual *pubertal *growth spurt.

## Results

A database was collected with longitudinal data of standardized shoe sizes of 636 girls and 513 boys. The individual series of ages of the clients ranged from 10 months to 17.1 years in girls, and 10 months to 17.2 years in boys.

After selection, the database provided 242 girls and 104 boys. Mean follow up time after 8 years of age was 5.4 (SD 1.2) years in girls, and 6.2 (SD 1.2) years in boys. The average number of measurements after 8 years of age was 8.9 (SD 1.9) in girls and 9.0 (SD 2.3) in boys (Table 2). Therefore, the average time between two visits after the age of 8 years was 0.61 years in girls (SD 0.29) and 0.69 years in boys (SD 0.34).

**Table 2:** General demographics of the used database.

	Girls (SD)	Boys (SD)	Total (SD)
Number of clients	242	104	346

Follow up time after age 8 in years	5.4 (1.2)	6.2 (1.2)	5.6 (1.2)

Number of measurements after age 8	8.9 (1.9)	9.0 (2.3)	8.9 (2.0)

SD = standard deviation

**Figure 2:**
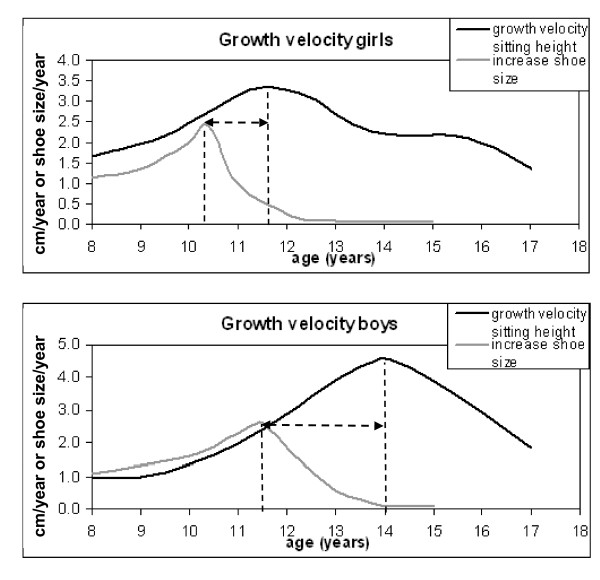
**Average growth curves of girls and boys from 8-18 years of age**. The differences between the timing of peak foot growth velocity based on shoe size and total body height are shown. Growth velocity of total body height is expressed in cm/year and increase in shoe size in shoe sizes/year, both on the same axis. Data concerning growth velocity of total body height are derived from Gerver and de Bruin with permission [5,6].

The average age of the peak increase in shoe size for girls was 10.4 years (SD 1.1), and for boys 11.5 years (SD 1.5). The average peak velocity of shoe size increase was 2.4 shoe sizes per year for girls (SD 1.3), and 2.6 shoe sizes per year for boys (SD 1.6). See Figure 2 and Table 3

**Table 3:** Results of database-study consisting longitudinal series of shoe sizes of 242 girls and 104 boys

	Girls (SD)	Boys (SD)	Total (SD)
Age at peak growth (years)	10.4 (1.1)	11.5 (1.5)	10.7 (1.4)

Growth speed at time of peak growth (shoe sizes/year)	2.4 (1.3)	2.6 (1.6)	2.5 (1.4)

Age at start plateau-phase	12.1 (0.8)	13.7 (1.0)	12.4 (1.0)

Time between age at peak growth velocity and start of plateau-phase	1.8 (1.2)	2.3 (1.6)	1.9 (1.3)

Shoe size at plateau-phase	38.9 (1.5)	41.4 (1.8)	39.3 (1.8)

The average age for girls (n = 138) to reach a plateau phase in their shoe size was 12.0 years (SD 0.8). At that time the shoe size was on average 38.9 (SD 1.5). Boys (n = 36) reached a plateau at the age of 13.7 years (SD 1.0), with a shoe size of 41.1 (SD 1.8) (Table 3).

The average longitudinal curves for peak increase of the shoe size of girls and boys are shown in Figure 2.

## Discussion

Many researchers have tried to predict the timing of the pubertal growth spurt, or peak growth velocity of total body height in the individual patient. Less researchers have investigated the prediction of the growth spurt in spinal length or sitting height, but this knowledge is of great value for optimizing treatment strategies in patients with adolescent idiopathic scoliosis.

This study showed that the timing of the peak increase in shoe size was 10.4 years in girls, and 11.5 years in boys. Furthermore, the shoe size did not increase any further for at least 1 year after the age of 12.0 (SD 0.8) in girls, and 13.7 years (SD 1.0) in boys. Stavlas et al. also showed a different growth potential of the feet in boys and girls in the sense that the physiological process of foot development occurred earlier in girls in comparison to boys[13].

To determine the relationship between the timing of the peak increase in shoe size and the timing of the pubertal growth spurt in sitting height, a comparison was made to reliable growth data of a comparable Dutch population. Secular trends in the pubertal growth spurt should be taken into account when comparing present data with known data for sitting height. Therefore growth data from Gerver and de Bruin were taken which were collected from 1995 to 1999[5], in comparison to the shoe sizes in present study which were collected from 1991 to 2008. Gerver and De Bruin[5] found a peak growth velocity of sitting height to occur at 11.7 years in girls (SD 0.8), and at 14.0 years in boys (SD 0.9). Thus, the average peak increase in shoe size generally occurred 1.3 years and 2.5 years before the average peak growth of sitting height, in girls and boys respectively. A second useful finding in the present study is that on average, the increase in shoe size diminishes when the peak growth velocity of sitting height occurs. The plateau phase of shoe size began at 12 years in girls, and a little under 14 years in boys, nearly similar to when the growth velocity of sitting height in both groups is largest (Figure 2).

These results suggest that the longitudinal course in shoe size and the timing of the peak increase in shoe size can be helpful as a first indication for the timing of the pubertal growth spurt of sitting height. A major advantage of using shoe size as an alternative for actual foot length is that patients and their parents can recall when they bought new shoes, and what the size was. Therefore the clinician already has access to some longitudinal data at the first visit of the patient, which are missing for actual foot length. Furthermore, this is a very easy and non time consuming way of getting a first impression on the stadium of growth of the patient. It is important to ask for the course in shoe size. Children and their parents remember to a large extent that they first had to buy a larger size after 1 year and than suddenly they had to buy a larger size after 0.5 year. When the increase in shoe size is approximately 2.5 sizes per year (in both girls and boys), the physician knows that on average the peak growth velocity of sitting height will occur 1.3 or 2.5 years later in girls and boys respectively. A pilot study in the orthopaedic outpatient clinic revealed that from 20 patients and their parents, 16 knew the course of their shoe size for at least 1.5 years back. However, repeated measurements should be performed in a coming longitudinal study to reveal a possible recall bias.

Present results could not be compared with existing data since no studies were found who investigated the course of the shoe size in children. However, when comparing results of the present study with four different studies measuring actual foot length, striking agreements were seen[8,10,14,15] (Table 4). The range for the timing of peak growth velocity of foot length was 10.4 to 12.3 years in girls (with three studies between 10.4 and 10.6 years), and 12.1 to 13.8 years in boys. The agreements for girls were largest, whereas the boys in present study showed a somewhat earlier growth spurt in shoe size in comparison to the other studies. No conclusive explanation for the differences concerning the boys could be found.

**Table 4:** Comparison of the present results with previous studies

Study		Girls (SD)	Boys (SD)
Present study 2009	Participants	242	104

	PGV SS	10.4 (1.1)	11.5 (1.5)

	Cessation	12.1 (0.8)	13.7 (1.0)

Anderson et al 1956	Participants	20	20

	PGV FL	10.5	12.5

	Cessation	13.5	16.0

Cameron et al 1982	Participants	20	29

	PGV FL	12.3 (0.3)	13.8 (0.3)

	Cessation		

Liu et al 1998	Participants	198	146

	PGV FL	10.4 (0.8)	12.1 (0.9)

	Cessation	13.6 (1.2)	15.6 (1.3)

Welon et al 1979	Participants	215	121

	PGV FL	10.6	13.0

	Cessation		

Standard Deviations (SD) were not shown in all studies. PGV SS = age in years at peak foot growth velocity based on shoe size. PGV FL = age in years at peak growth velocity of foot length. Cessation = the age at which the increase in shoe size or the growth of foot length is finished. Not all studies showed the age of cessation of the growth.

Anderson et al[14] and Liu et al[15] found a cessation of the foot growth around 13.5 years in girls and 16 years in boys (Table 4). This age was higher compared to the present study for the reason that Anderson and Liu used a limit of less than 2 mm growth of the feet. One shoe size represents approximately 7 mm, so the feet can still grow more than 2 mm before a larger shoe size is needed. There might be additional factors to explain the difference of 2.3 years in boys. This will be discussed further in the next section.

A disadvantage of the initial available database was that less data were available for higher ages. Many clients only had measurements until age 10 or 11. As a result of the strict selection criteria, the initial group of clients was therefore decreased by respectively 62% and 80% for girls and boys. However, no indications were found in the non-selected group for differences in increases of shoe size. Furthermore, as will be outlined below, the results were unaffected by changing the selection criteria, thereby suggesting that the results were not biased by selection.

The minimum age for the last measurement was higher for boys and therefore fewer boys were included than girls. The minimum age was chosen to prevent the inclusion of "false" growth spurts, as described earlier in the methods section. It was shown during the analysis of the data that the ages of the peak increase did not change when only the clients were selected who had a last measurement above a higher age. For example, when only the male clients were selected who had the last measurement of shoe size above 14 years of age (instead of 13) the peak growth velocity still occurred at 11.5 years (n = 55). When the group was selected who had their last measurement above 15 years (n = 27), the age at peak growth velocity was 11.4 years. A similar test was done for the female clients and 12 years appeared to be an adequate cut-off point (n = 131 for the group with the last measurement above age 13 and age at peak growth velocity 10.4 years; n = 50 for the group with the last measurement above age 14 and age at peak growth velocity 10.6 years). Thus, for both boys and girls, the primary outcome measure, i.e. the age of peak foot growth velocity based on shoe size, seems reliable.

This study showed a plateau phase in shoe size of at least 1 year in boys starting on average at 13.7 years. The shoe size at that time was on average 41.4, which was smaller than expected.

**Figure 3:**
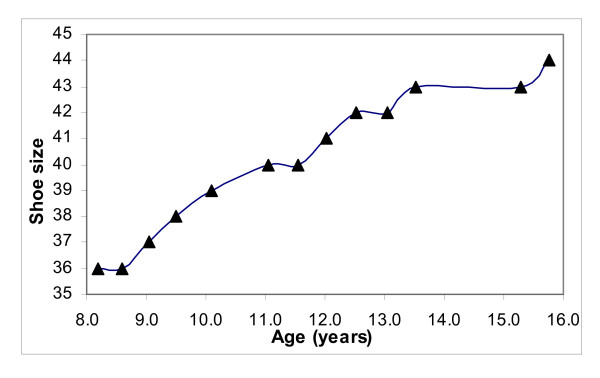
**Example of a growth curve of shoe size of a boy**. The curve shows a plateau phase between 13.5 and 15.3 years and again an increase of the shoe size after 15 years. Shoe size is expressed in European standards.

A possible explanation could be that more remaining growth of the feet occurred in boys at a higher age. Not all boys with data at higher ages showed a plateau phase. Boys without a plateau were not included in the selection of the boys for the data analysis of the plateau phase. However, these boys possibly showed a plateau phase at a later age with a larger shoe size, but these data were not available in the present database. The database provided only few boys who had longitudinal data after the age of 15 or 16. One example showed a plateau phase from 13.5 to 15.3 years, and again some growth of the shoe size after that (Figure 3). It is possible that some boys have a "second" short growth spurt of shoe size in a later stage of puberty. Therefore, we may have underestimated the secondary outcome measures, i.e. final shoe size and the age of cessation of foot growth, in boys.

There are some limitations to the use of the shoe size. Different brands do not always use similar size-length ratios. E.g. size 39 in one brand is not necessarily the same length as size 39 in a different brand. Pilot work for the present study showed that clients recognize this difference and know their "general" shoe size. However, further research should be performed to evaluate the reliability of the use of shoe size in a clinical setting and to evaluate a possible recall bias of patients and their parents.

For further validation of the use of shoe size for prediction of the peak growth velocity of sitting height it is essential to combine both measurements in a single study. Present work of our study group will concern these measurements, but the results will be available only after collection of longitudinal data for several years[16].

## Conclusions

The present data suggest that the course of the shoe size of children visiting the outpatient clinic can be useful as a first indicator for the timing of the pubertal growth spurt of sitting height. This claim needs verification by direct comparison of individual shoe size and sitting height data. If such work supports our claim, a step forward can be made in clinical decision making regarding adolescent idiopathic scoliosis.

## Competing interests

The authors declare that they have no competing interests.

## Authors' contributions

All authors have read and approved the manuscript and believe the manuscript represents honest work. All authors (IB, IK, GJV, FHW, SKB, and AGV) have made substantial contributions to the design of the presented study. IB and IK have performed the data-analysis. IB has written the first manuscript. IK, FHW, SKB, GJV, and AGV have performed the reviewing of the manuscript.
